# COVID-19 Modulates Inflammatory and Renal Markers That May Predict Hospital Outcomes among African American Males

**DOI:** 10.3390/v13122415

**Published:** 2021-12-02

**Authors:** Wendy Fonseca, Nobuhiro Asai, Kazuma Yagi, Carrie-Anne Malinczak, Gina Savickas, Christine C. Johnson, Shannon Murray, Edward M. Zoratti, Nicholas W. Lukacs, Jia Li, Charles F. Schuler IV

**Affiliations:** 1 Department of Pathology, University of Michigan, Ann Arbor, MI 48109, USA; wfaguila@med.umich.edu (W.F.); nobua@med.umich.edu (N.A.); kyagi@med.umich.edu (K.Y.); carrieam@med.umich.edu (C.-A.M.); nlukacs@med.umich.edu (N.W.L.); 2 Translational and Clinical Research Center, Department of Internal Medicine, Henry Ford Hospital, Detroit, MI 48202, USA; gsavick1@hfhs.org (G.S.); smurray6@hfhs.org (S.M.); 3 Department of Public Health Sciences, Henry Ford Hospital, Detroit, MI 48202, USA; cjohnso1@hfhs.org (C.C.J.); jli4@hfhs.org (J.L.); 4 Division of Allergy and Immunology, Department of Internal Medicine, Henry Ford Hospital, Detroit, MI 48202, USA; ezoratt1@hfhs.org; 5 Mary H. Weiser Food Allergy Center, University of Michigan, Ann Arbor, MI 48109, USA; 6 Division of Allergy and Clinical Immunology, Department of Internal Medicine, University of Michigan, Ann Arbor, MI 48109, USA

**Keywords:** COVID-19, SARS-CoV-2, cytokines, renal toxicity

## Abstract

Background and Objectives: African Americans and males have elevated risks of infection, hospitalization, and death from SARS-CoV-2 in comparison with other populations. We report immune responses and renal injury markers in African American male patients hospitalized for COVID-19. Methods: This was a single-center, retrospective study of 56 COVID-19 infected hospitalized African American males 50+ years of age selected from among non-intensive care unit (ICU) and ICU status patients. Demographics, hospitalization-related variables, and medical history were collected from electronic medical records. Plasma samples collected close to admission (≤2 days) were evaluated for cytokines and renal markers; results were compared to a control group (*n* = 31) and related to COVID-19 in-hospital mortality. Results: Among COVID-19 patients, eight (14.2%) suffered in-hospital mortality; seven (23.3%) in the ICU and one (3.8%) among non-ICU patients. Interleukin (IL)-18 and IL-33 were elevated at admission in COVID-19 patients in comparison with controls. IL-6, IL-18, MCP-1/CCL2, MIP-1α/CCL3, IL-33, GST, and osteopontin were upregulated at admission in ICU patients in comparison with controls. In addition to clinical factors, MCP-1 and GST may provide incremental value for risk prediction of COVID-19 in-hospital mortality. Conclusions: Qualitatively similar inflammatory responses were observed in comparison to other populations reported in the literature, suggesting non-immunologic factors may account for outcome differences. Further, we provide initial evidence for cytokine and renal toxicity markers as prognostic factors for COVID-19 in-hospital mortality among African American males.

## 1. Introduction

Severe acute respiratory syndrome-coronavirus-2 (SARS-CoV-2) is the β-coronavirus responsible for the COVID-19 global pandemic, causing millions of infections and deaths (according to the WHO). COVID-19 causes varied clinical manifestations, from mild forms to pneumonia and acute respiratory distress syndrome (ARDS) [1,2,3]. Three COVID-19 severity phenotypes are described: “mild” (80%) with minor symptoms not progressing to severe disease, “moderate” (15%) requiring hospitalization, and “severe” (5%) requiring critical care management and at risk of a fatal outcome [4,5].

The immune reaction to SARS-CoV-2 infection can lead to cytokine storm syndrome, with substantial mortality risk [6,7]. Inflammatory mediators, such as interleukin (IL) IL-2, IL-7, IL-10, IL-1, IL-6, IL-18, IL-33, tumor necrosis factor (TNF), monocyte chemoattractant protein-1 (MCP-1; also known as CCL2), macrophage inflammatory protein 1 alpha (MIP-1α; also known as CCL3), CXC-chemokine ligand 10 (CXCL10), RANTES (also known as CCL5), C-reactive protein (CRP), ferritin, and D-dimer, are upregulated systemically in severe COVID-19 cases [6,7]. Anti-inflammatory treatments targeting IL-6 and IL-1, as well as hemoperfusion techniques, have shown evidence of utility in severe COVID-19, suggesting an exacerbated inflammatory response partly mediates severity [8,9,10]. SARS-CoV-2 can therefore have systemic effects [11,12]; for example, renal disease is also associated with in-hospital mortality in COVID-19 [12,13]. Renal failure for severe COVID-19 may be multifactorial as the virus causes cytokine storm, endothelial dysfunction, hypercoagulability, and/or sepsis [5,12,14].

The Centers for Disease Control and Prevention (CDC) has reported that sex as well as race and ethnicity are risk factors for COVID-19 rates and severity via factors including socioeconomic status, healthcare access, co-morbidities, and occupational and environmental exposure [15]. Males in particular have an elevated mortality risk [9]. In the United States (US), the pandemic has disproportionately affected minority communities, particularly African Americans [16]; reports suggest the African American population has up to 1.4× the rate of SARS-CoV-2 infection, 3.7× the rate of hospitalization, and 2.8× the rate of mortality versus white, non-Hispanic, or Latino persons (CDC). In the state of Michigan, USA, African Americans represent 14% of the state population but up to 37% of the positive COVID-19 cases and 42% of COVID-19 deaths [15,17,18]. The African American community’s disproportionate disease severity has been attributed to race, socioeconomic, and health disparities [17,18]; however, African American mortality is no worse than other groups once admitted to the hospital [19,20,21].

In this work, we aimed to understand how SARS-CoV-2 infection and inflammation systemically affects older African American males by studying clinical characteristics, the systemic immune response, and renal toxicity markers in a single-center retrospective cohort study of hospitalized COVID-19 patients.

## 2. Materials and Methods

### 2.1. Ethics Approval

All human studies were performed following University of Michigan (approval HUM00180532) and Henry Ford Health System (approval 13297) institutional review board-approved protocols. All participants provided written informed consent prior to specimen collection and enrollment in the Henry Ford Health System COVID-19 biobank. The institutional review board at Henry Ford Health System approved the biospecimen collection protocol and banking. Subsequently, samples requested for this project and accompanying clinical information were evaluated in a de-identified manner.

### 2.2. Study Design and Patients

From the start of COVID-19 in March 2020 in Michigan through September 2020, over 11,000 patients were admitted with COVID-19 to Henry Ford Health System (HFHS) in Southeast Michigan. A cohort of over 2000 patients were enrolled in the Translational and Clinical Research Center Biobank within HFHS. For this single-center, retrospective cohort study, we selected African American male patients ≥ 50 years old, admitted for the first time to HFHS for COVID-19 infection from March to September 2020. Participants were admitted to the general or intensive care units (ICU). Patients on the general floor or ICU who were not ventilated at baseline but progressed to need a ventilator or who were stepped up in care to the ICU during their stay were excluded. COVID-19 status was defined by clinical nucleic acid amplification testing of nasopharyngeal swabs positive for SARS-CoV-2. No asymptomatic patients were included. The control cohort consisted of a convenience sample of 31 non-COVID-19 patients (inpatient or Emergency Department) encountered during the same time period with negative clinical nucleic acid amplification testing of nasopharyngeal swabs for COVID-19 with the same demographic characteristics; patients with a respiratory illness were excluded from this group, as were those who were presenting with an infectious disease.

Baseline patient characteristics collected included age, sex, past medical history, and medications. COVID-19-related symptoms and admission respiratory status were collected. Clinical outcomes collected included admission duration, level of care, respiratory care needs including mechanical ventilation status, COVID-19-related medications, and in-hospital mortality. The earliest banked blood sample after hospital admission was used for all analyses. Median time between admission and blood drawn was <1 day; interquartile range was 0–3 days.

### 2.3. Multi-Analyte Immunoassay and Enzyme-Linked Immunosorbent Assay (ELISA)

Cytokine levels of human IL-6, IL-18, MCP-1, MIP-1α, RANTES, TNF-α, IFN-β, IFN-α2, and IFN-γ were analyzed using a commercially available multi-analyte immunoassay (Bio-Plex cytokine assay, Bio-Rad Laboratories, Hercules, CA, USA). For the detection of IL-33 and IL-1β, the R&D Duo set ELISA kit (R&D Systems, Minneapolis, MN, USA) was used per the manufacturer’s instructions. Renal toxicity markers were evaluated using a commercial equipment Bio-Plex Pro RBM Human Kidney Toxicity Assays (Bio-Rad, Hercules, CA, USA) to measure glutathione S-transferase (GST), osteopontin, trefoil factor 3 (TFF3), neutrophil gelatinase-associated lipocalin (NGAL), calbindin, clusterin, kidney injury molecule-1 (KIM-1), and beta-2 microglobulin (B2M), following the manufacturer’s instructions.

### 2.4. Statistical Analysis

Patient characteristics were summarized using standard descriptive statistics: mean/standard deviation for continuous variables and frequency for categorical variables. To compare between the groups, we used the nonparametric Wilcoxon rank sum test for continuous variables and the Fisher’s exact test for categorical variables.

Cytokines, in which more than 50% of samples were below the detection limit, were not included in the analysis. Distributions of the cytokine values were assessed and log2 transformed to render parametric statistical analyses. To compare cytokine/renal toxicity marker levels between groups, we performed two-sample *t*-tests or ANOVA. A sensitivity analysis based on nonparametric tests (Wilcoxon rank sum test or Kruskal–Wallis tests) was also conducted.

Additionally, we estimated the association between cytokine/renal toxicity markers with COVID-19 outcomes using the Firth logistic regression. Due to the small sample size and events, the ordinary logistic regression that is based on maximum likelihood estimation will yield biased estimates of coefficients. A penalized version such as Firth logistic regression is generally recommended for small-sample studies. Odds ratios of outcomes with a fold increase in marker levels were estimated. ORs were estimated on the basis of unadjusted and adjusted models. To correct for multiple testing issues, we adjusted *p*-values using the Benjamini–Hochberg approach for all above analyses. Adjusted *p*-values (FDR: false discovery rate) less than 0.1 or 0.05 are considered as suggestive cutoffs.

We next explored the potential of cytokines and renal toxicity markers as a complement variable for COVID-19 outcome risk stratification. Missing values for cytokines due to detection limit were imputed using quantile regression imputation of left-censored data before the analysis. The elastic-net logistic regression (ENET) was used to construct classifiers for in-hospital mortality. The tuning parameters of ENET were determined on the basis of the internal fivefold cross validation (CV). The tuning parameter limits the size of the coefficients and can result in models with small number of variables. The results were compared for a model based on patients’ demographics and comorbidity, as well as a model based on patients’ demographics, comorbidity, cytokines, and renal toxicity markers. The prediction metrics for comparison included receiver operating curves (ROC) and precision-recall curves (PRC). The whole process was evaluated by a repeated fivefold CV. The goal is to test the model’s ability to predict new data, to flag problems such as overfitting and selection bias, and to provide an insight on how the model will generalize to an independent external data set.

## 3. Results

### 3.1. Patient Cohort

A total of 56 COVID-19-affected and 31 non-COVID-19-affected African American male patients were analyzed (Table 1). Of those with COVID-19, 30 cases were treated in the ICU and 26 were hospitalized without requiring ICU care. ICU status patients had higher admission qSOFA scores. A total of 48% of COVID-19 patients were between the ages of 61 and 70. A total of 57% of COVID-19 patients had a history of smoking, and these patients were less likely to have circulatory/vascular disease or a cancer history. More substance abuse and smoking were seen in non-COVID-19 patients. Four patients were on IL-6 cytokine inhibitors; these were excluded for the analysis of IL-6.

Patient characteristics and comorbidities were compared for in-hospital mortality among COVID-19 patients (Table 2). When comparing patient characteristics with in-hospital mortality, we found that higher qSOFA was associated with in-hospital mortality. More patients were on dialysis in the in-hospital mortality group (Table 2).

### 3.2. Association of Cytokines and Renal Injury Markers with Demographics and Baseline Comorbidities

**Figure 1:**
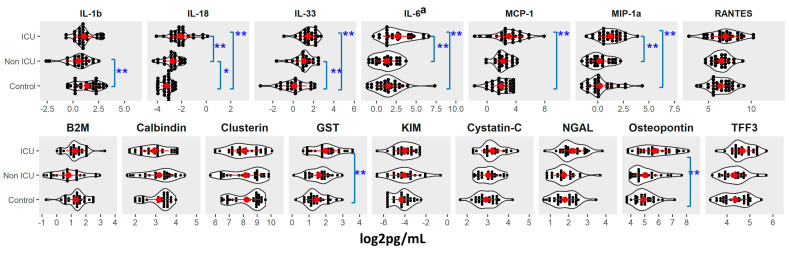
Cytokine and Renal toxicity marker levels by COVID-19 status at sample collection. *: adjusted *p*-value (FDR) ≤ 0.1; **: adjusted *p*-value (FDR) ≤ 0.05. a: patients on cytokine inhibitor were excluded for analysis. Details were provided in Table S1.

We detected increases in IL-18, IL-6, and MIP-1α in the COVID-19 ICU group versus controls and the non-ICU group (FDR < 0.05; Figure 1 and Table S1). We also detected an increase in IL-33 and MCP-1 in the COVID-19 ICU group over controls (FDR < 0.05; Figure 1 and Table S1). Cytokines IL-18 and IL-33 were higher and IL-1β was lower in COVID-19 non-ICU patients versus controls (FDR < 0.1 or < 0.05). We also detected elevated GST and osteopontin in the ICU group versus controls (FDR < 0.05, Figure 1). COVID-19 patients who had a genitourinary disorder had higher MCP-1 levels (FDR < 0.05; Figure S1). IL-1β levels were lower in COVID-19 patients with neurological disorders (FDR < 0.05; Figure S1). There were no significant associations with age or BMI.

We detected elevated osteopontin and GST in samples from COVID-19 ICU patients versus controls (FDR < 0.05; Figure 1). COVID-19 patients who had dialysis or genitourinary disorder had higher levels of osteopontin, GST, and TFF3 (FDR < 0.1 or <0.05; Figure S2).

### 3.3. Association between Cytokines, Renal Injury Markers, and Risk of COVID-19 in-Hospital Death

**Figure 2:**
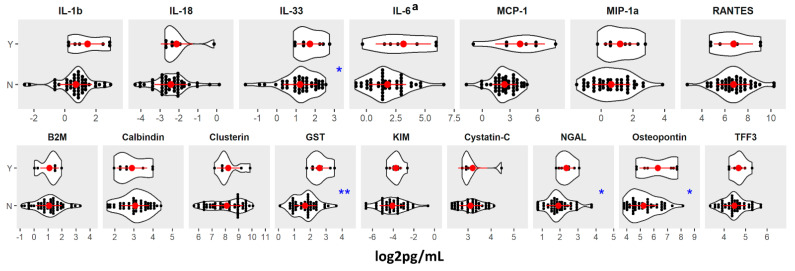
Cytokine and Renal toxicity marker levels by COVID-19 in-hospital death (Y vs. N). *: adjusted *p*-value (FDR) ≤ 0.1; **: adjusted *p*-value (FDR) ≤ 0.05. a: patients on cytokine inhibitor were excluded for analysis.

IL-33 was significantly higher in in-hospital non-survivors compared to survivors (FDR < 0.1; Figure 2). GST, NGAL, and osteopontin were significantly elevated in non-survivors as well (FDR < 0.1 or <0.05; Figure 2).

**Figure 3:**
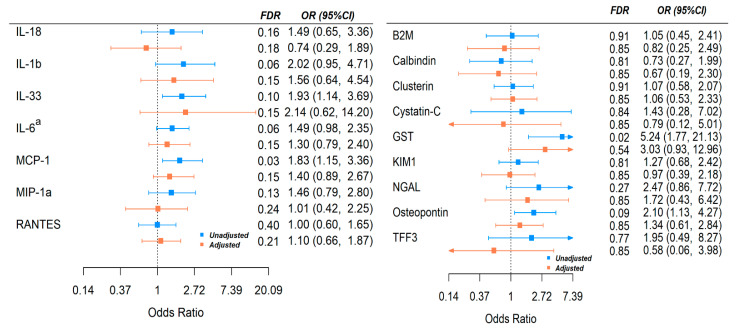
Association of Cytokines with risk of COVID-19 in-hospital mortality before and after adjustment for potential confounders. Adjusted model: dialysis, qSOFA. OR: odds ratio of outcome with 1-fold increase in cytokine levels. a: patients on cytokine inhibitor were excluded for analysis.

We further estimated the odds ratio of in-hospital death with onefold increase in the marker levels from an unadjusted and adjusted model (see the Methods section). In the adjusted model, qSOFA score and dialysis were adjusted as potential confounders (time between blood draw and admission was also considered but did not change the conclusion). IL-6, MCP-1, and IL-33 were significantly associated with risk of in-hospital mortality; the results are marginal after adjustment for other variables (Figure 3). For renal injury markers, higher GST and osteopontin levels were associated with higher risk of in-hospital death, although again the results were marginal after adjustment for other variables (Figure 3).

**Figure 4:**
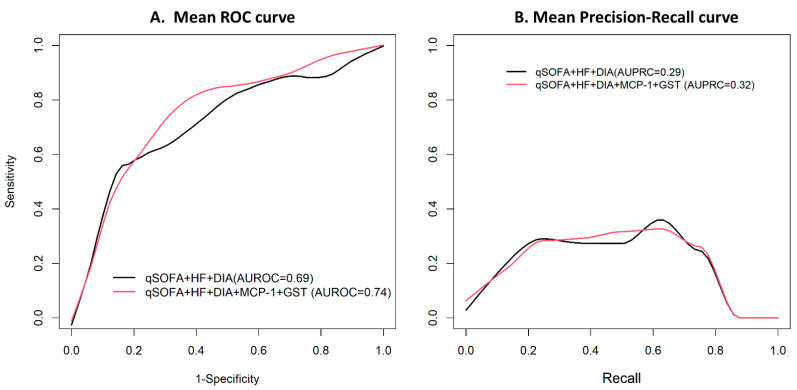
Comparison of prediction accuracy for clinical variable-based model versus cytokine and renal toxicity-based model for predicting in-hospital death in patients with COVID-19. qSOFA: qSOFA ≥ 1 vs. <1; HF: heart failure; DIA: dialysis.

We further considered using cytokine and renal injury markers to complement risk stratification. The ENAT model selected qSOFA score, history of heart failure, and dialysis as important clinical risk factors for in-hospital mortality. The area under the ROC (AUROC) was 0.69 (95% CI: 0.47–0.91; Figure 4). In addition to clinical factors, MCP-1 and GST were independently predictive of patient in-hospital mortality; the AUROC of the secondary model was 0.74 (95% CI: 0.56–0.92). It was significantly improved, and the *p*-value was found to be 0.038, obtained by parametric bootstrap. Precision-recall curve was also higher when MCP-1 and GST were included compared to a clinical risk factor-only model (0.32 vs. 0.29; *p*-value = 0.006).

## 4. Discussion

SARS-CoV-2, the virus responsible for COVID-19, has caused a worldwide pandemic with millions of cases and deaths (WHO). An overwhelming systemic inflammatory response leading to multi-organ failure is associated with mortality and severe disease [6,7]. Males have an elevated mortality risk [9]. African Americans have been adversely impacted by this pandemic, with disproportionate hospitalizations and deaths, particularly early on in the pandemic timeline [22,23,24,25]. Evidence has emerged that African Americans do not have elevated in-hospital mortality over other groups once admitted to the hospital [18]. However, African American males have not been studied extensively to examine systemic inflammatory responses to COVID-19. We sought to define the systemic inflammatory response in this highest risk group and evaluate whether systemic inflammation and renal dysfunction markers described in previous reports could be applied to this population.

In this study, we observed key similarities in the systemic inflammatory response to prior reports. IL-6 and IL-18, among others, were elevated in ICU status COVID-19 patients versus non-ICU COVID-19 patients and hospitalized controls [9,26,27]. IL-33, MCP-1/CCL2, and MIP-1α/CCL3 were also elevated in ICU status COVID-19 subjects over controls, consistent with other reports [28,29,30,31]. Interestingly, a clinical study in China showed that a similar set of cytokines and chemokines were associated with COVID-19 severity; levels of IL-6, IL-7, IL-10, IL-18, G-CSF, M-CSF, MCP-1, MCP-3, IP-10, MIG, and MIP-1α were more upregulated in males than females [32]. A Norwegian study reported increased IL-6 and MCP-1 were associated with respiratory failure in COVID-19-positive patients [33]. Many of these cytokines, including IL-6, IL-18, IL-33, MCP-1, and MIP-1α, were elevated in the COVID-19 ICU and/or in-hospital mortality groups in our study [34].

Renal dysfunction is associated with in-hospital mortality in COVID-19 [12,13]. We evaluated whether markers of renal dysfunction were associated with ICU status and/or in-hospital mortality in the present study. GST and osteopontin were associated with COVID-19 ICU status; GST, NGAL, and osteopontin were elevated among the in-hospital mortality group. NGAL has been implicated in COVID-19-related in-hospital mortality previously [35]. A retrospective COVID-19 study from the University of Tokyo Hospital of 17 critically ill patients described upregulation in urinary NGAL levels in patients that developed acute kidney injury [36], suggesting the increased levels of renal toxicity markers in our study could be an indicator of future development of acute kidney injury and possibly related with systemic organ failure. In addition, GST is involved in cellular detoxification and neutralization of oxidative stress; it has been reported that two isoenzymes, GSTT1-1 and GSTM1-1, are genetically deleted in a high percentage of the human population, with significant ethnic differences [37]. A correlation of GSTT1 polymorphism and COVID-19 outcome has been suggested, although no clinical data have been presented to correlate these results [38]. These findings around NGAL, GST, and osteopontin in this population deserve further study, as they may correlate with renal dysfunction and/or in-hospital mortality in COVID-19 and might enhance detection of such dysfunction at early stages.

The present study has limitations. First, this was a single-center study, which limits the broad applicability without further research in larger groups. Second, we enrolled an all-male cohort; while this was done to study an under-evaluated, specific population disproportionately affected by the COVID-19 pandemic and remove variation in support of statistical power, this could limit broad applicability. The control group that was enrolled included hospitalized and emergency department patients and did not include patients with a respiratory illness; while this was done to provide a clear baseline value for cytokine and other measurements, this may limit the applicability of the study. Last, while we attempted to use plasma cytokines and renal toxicity markers as prognostic factors, the study was hindered by too small of a sample size to provide precise estimates of association and model predictability. Validation of these findings is needed in a larger-scale study.

In conclusion, this study restricted to African American male patients hospitalized with COVID-19 showed key qualitative similarities in the systemic inflammatory response to COVID-19 to other populations worldwide. In addition, the results suggest novel markers of renal dysfunction that may predict in-hospital mortality from COVID-19. While the African American population has suffered disproportionately from COVID-19, this study suggests that the pandemic’s impact may not emanate from differences in the type of overall systemic inflammatory responses but perhaps from other factors that sort according to race and/or ethnicity, such as socioeconomics, occupational or other exposure patterns, or comorbidities. The potential predictive capacity of plasma cytokine and renal toxicity markers might be generalizable if confirmed in additional cohorts and could add to the existing predictive models being developed for COVID-19 mortality to help better understand mortality mechanisms in this disease.

## Figures and Tables

**Table 1:** Study participant characteristics by COVID-19 status.

		Control	Non-ICU	ICU	*p*
		(N = 31)	(N = 26)	(N = 30)	
BMI	≤30	11 (35.5%)	10 (38.5%)	14 (46.7%)	0.678
	>30	20 (64.5%)	16 (61.5%)	16 (53.3%)	
Smoke	Never/unknown	8 (25.8%)	15 (50.0%)	9 (34.6%)	0.025
	Former smoker	8 (25.8%)	11 (36.7%)	12 (46.2%)	
	Current smoker	15 (48.4%)	4 (13.3%)	5 (19.2%)	
Substance abuse	Yes	14 (45.2%)	6 (23.1%)	4 (13.3%)	0.022
	No	17 (54.8%)	20 (76.9%)	26 (86.7%)	
Age	50–60	11 (35.5%)	8 (30.8%)	7 (23.3%)	0.624
	61–70	14 (45.2%)	10 (38.5%)	17 (56.7%)	
	>70	6 (19.4%)	8 (30.8%)	6 (20.0%)	
qSOFA	<1	NA	16 (61.5%)	4 (13.3%)	<0.001
	≥1	NA	10 (38.5%)	26 (86.7%)	
	Medical History				
Cardiovascular and thoracic	Yes	30 (96.8%)	24 (92.3%)	28 (93.3%)	0.735
	No	1 (3.2%)	2 (7.7%)	2 (6.7%)	
Hypertension	Yes	28 (90.3%)	24 (92.3%)	26 (86.7%)	0.827
	No	3 (9.7%)	2 (7.7%)	4 (13.3%)	
Heart failure	Yes	10 (32.3%)	5 (19.2%)	9 (30.0%)	0.57
	No	21 (67.7%)	21 (80.8%)	21 (70.0%)	
Respiratory and pulmonary	Yes	13 (41.9%)	6 (23.1%)	16 (53.3%)	0.068
	No	18 (58.1%)	20 (76.9%)	14 (46.7%)	
G.I./digestive	Yes	16 (51.6%)	8 (30.8%)	12 (40.0%)	0.29
	No	15 (48.4%)	18 (69.2%)	18 (60.0%)	
Endocrine	Yes	17 (54.8%)	21 (80.8%)	16 (53.3%)	0.064
	No	14 (45.2%)	5 (19.2%)	14 (46.7%)	
T2D	Yes	14 (45.2%)	19 (73.1%)	14 (46.7%)	0.075
	No	17 (54.8%)	7 (26.9%)	16 (53.3%)	
Thyroid	Yes	2 (6.5%)	2 (7.7%)	3 (10.0%)	0.891
	No	29 (93.5%)	24 (92.3%)	27 (90.0%)	
Neurological	Yes	14 (45.2%)	12 (46.2%)	6 (20.0%)	0.06
	No	17 (54.8%)	14 (53.8%)	24 (80.0%)	
Genitourinary	Yes	22 (71.0%)	20 (76.9%)	25 (83.3%)	0.538
	No	9 (29.0%)	6 (23.1%)	5 (16.7%)	
Dialysis	Yes	24 (77.4%)	22 (84.6%)	21 (70.0%)	0.432
	No	7 (22.6%)	4 (15.4%)	9 (30.0%)	
Circulatory and vascular	Yes	23 (74.2%)	14 (53.8%)	12 (40.0%)	0.025
	No	8 (25.8%)	12 (46.2%)	18 (60.0%)	
Cancer history	Yes	5 (16.1%)	1 (3.8%)	0 (0%)	0.036
	No	26 (83.9%)	25 (96.2%)	30 (100%)	

**Table 2:** COVID-19 patient characteristics by in-hospital mortality.

		Hospital Death		
		Yes	No	
		(N = 8)	(N = 48)	
BMI	≤30	5 (63%)	27 (56%)	>0.999
	>30	3 (38%)	21 (44%)	
Smoke	Never/unknown	4 (50%)	20 (42%)	0.54
	Former smoker	4 (50%)	19 (40%)	
	Current smoker	0 (0%)	9 (19%)	
Substance abuse	Yes	2 (25%)	8 (17%)	0.623
	No	6 (75%)	40 (83%)	
Age	50–60	1 (13%)	14 (29%)	0.478
	61–70	4 (50%)	23 (48%)	
	>70	3 (38%)	11 (23%)	
qSOFA	<1	0 (0%)	20 (42%)	0.041
	≥1	8 (100%)	28 (58%)	
Charleston comorbidity index	1–2	0 (0%)	6 (13%)	0.588
	3–4	1 (13%)	10 (21%)	
	>4	7 (88%)	32 (67%)	
Medical History				
Cardiovascular and thoracic	Yes	8 (100%)	44 (92%)	>0.999
	No	0 (0%)	4 (8%)	
Hypertension	Yes	7 (88%)	43 (90%)	>0.999
	No	1 (13%)	5 (10%)	
Heart failure	Yes	0 (0%)	14 (29%)	0.18
	No	8 (100%)	34 (71%)	
Respiratory and pulmonary	Yes	4 (50%)	18 (38%)	0.698
	No	4 (50%)	30 (63%)	
G.I./digestive	Yes	2 (25%)	18 (38%)	0.697
	No	6 (75%)	30 (63%)	
Endocrine	Yes	6 (75%)	31 (65%)	0.703
	No	2 (25%)	17 (35%)	
T2D	Yes	4 (50%)	29 (60%)	0.704
	No	4 (50%)	19 (40%)	
Thyroid	Yes	2 (25%)	3 (6%)	0.144
	No	6 (75%)	45 (94%)	
Neurological	Yes	1 (13%)	17 (35%)	0.414
	No	7 (88%)	31 (65%)	
Genitourinary	Yes	7 (88%)	38 (79%)	>0.999
	No	1 (13%)	10 (21%)	
Dialysis	Yes	5 (63%)	8 (17%)	0.012
	No	3 (38%)	40 (83%)	
Circulatory and vascular	Yes	3 (38%)	23 (48%)	0.712
	No	5 (63%)	25 (52%)	
Cancer history	Yes	0 (0%)	1 (2%)	>0.999
	No	8 (100%)	47 (98%)	
Symptoms				
SOB	Yes	7 (88%)	30 (65%)	0.411
	No	1 (13%)	16 (35%)	
Cough	Yes	4 (50%)	26 (57%)	>0.999
	No	4 (50%)	20 (43%)	
Fever/chills	Yes	6 (75%)	22 (48%)	0.253
	No	2 (25%)	24 (52%)	
Headache	Yes	0 (0%)	3 (7%)	>0.999
	No	8 (100%)	43 (93%)	
Nausea/diarrhea/vomiting	Yes	3 (38%)	15 (33%)	>0.999
	No	5 (63%)	31 (67%)	
Congestion	Yes	0 (0%)	2 (4%)	>0.999
	No	8 (100%)	44 (96%)	
Fatigue/body ache/weakness	Yes	7 (88%)	19 (42%)	0.024
	No	1 (13%)	26 (58%)	
Change in scent/smell/lack of appetite	Yes	0 (0%)	9 (20%)	0.324
	No	8 (100%)	36 (80%)	
Experimental/Relevant Medications				
Plaquenil/HCQ	Yes	8 (100%)	32 (68%)	0.091
	No	0 (0%)	15 (32%)	
Remdesivir	Yes	0 (0%)	2 (4%)	>0.999
	No	8 (100%)	43 (96%)	
Steroids	Yes	8 (100%)	35 (74%)	0.178
	No	0 (0%)	12 (26%)	
Azithromycin	Yes	1 (13%)	12 (26%)	0.664
	No	7 (88%)	35 (74%)	
Cytokine Inhibitors	Yes	2 (25%)	3 (6%)	0.149
	No	6 (75%)	44 (94%)	
NSAIDS	Yes	2 (25%)	14 (30%)	>0.999
	No	6 (75%)	33 (70%)	

## Data Availability

The data presented in this study are available on request from the University of Michigan and Henry Ford Health System Data Offices. The data are not publicly available due to ethical reasons.

## Supplementary Materials

The following are available online at https://www.mdpi.com/article/10.3390/v13122415/s1, Figure S1: Cytokine levels by COVID-19 patient characteristics at baseline, Figure S2. Renal toxicity levels by COVID-19 patient characteristics at baseline, Table S1. Distribution of cytokine, renal toxicity marker levels by COVID status.
