# Supplementary material for: COVID-19 Modulates Inflammatory and Renal Markers That May Predict Hospital Outcomes among African American Males
Source: Viruses. 2021 Dec 2;13(12):2415. doi: 10.3390/v13122415 (PMC8708877; doi:10.3390/v13122415)
Supplement: Supplementary file 1 [file viruses-13-02415-s001.zip › viruses-1355348-supplementary.pdf]

Table S1. Distribution of cytokine, renal toxicity marker levels (median [IQR] of log2pg/mL) by COVID status.

	Control	Non ICU	ICU	p-value			FDR		
	(N=31)	(N=26)	(N=30)	ICU vs control	Non-ICU vs control	ICU vs non-ICU	ICU vs control	Non-ICU vs control	ICU vs non-ICU
**IL6**	1.41 [0.438, 2.36]	1.30 [0.184, 2.35]	2.29 [1.40, 4.14]	0.021	0.872	0.007	0.037	0.994	0.016
**IL18**	-3.18 [-3.47, -3.00]	-2.69 [-2.92, -2.31]	-2.15 [-2.60, -1.94]	<0.001	0.030	0.002	<0.001	0.071	0.011
**MCP1**	2.02 [1.18, 3.10]	2.08 [1.55, 2.84]	3.06 [2.19, 4.28]	0.027	0.824	0.138	0.038	0.994	0.193
**MIP1a**	-0.05 [-0.60, 0.54]	-0.04 [-0.49, 0.91]	1.19 [0.524, 1.94]	0.004	0.981	0.003	0.009	0.994	0.011
**RANTES**	6.44 [5.60, 7.30]	6.50 [5.85, 7.09]	7.04 [6.20, 8.17]	0.287	0.994	0.372	0.335	0.994	0.372
**IL1b**	1.51 [0.342, 2.24]	0.67 [0.07, 1.22]	0.948 [0.59, 1.38]	0.492	0.008	0.126	0.492	0.028	0.193
**IL33**	0.23 [-0.64, 0.77]	1.10 [0.764, 1.55]	1.50 [1.10, 2.10]	<0.001	0.004	0.296	<0.001	0.028	0.345
**Calbindin**	3.36 [2.81, 3.57]	3.39 [2.69, 3.66]	2.98 [2.35, 3.33]	0.418	0.998	0.421	0.603	0.998	0.541
**Clusterin**	8.87 [7.12, 9.07]	8.80 [6.88, 9.01]	7.95 [7.23, 8.96]	0.796	0.975	0.914	0.896	0.998	0.966
**GST**	1.31 [1.08, 1.75]	1.75 [1.08, 1.94]	1.94 [1.45, 2.41]	0.008	0.659	0.104	0.037	0.998	0.188
**KIM1**	-4.32 [-5.06, -3.74]	-4.06 [-5.06, -3.47]	-4.06 [-4.64, -3.32]	0.469	0.659	0.966	0.603	0.998	0.966
**B2M**	1.42 [1.00, 1.64]	0.69 [0.08, 1.38]	1.24 [1.03, 1.69]	0.985	0.049	0.074	0.985	0.440	0.166
**Cystatin_C**	2.94 [2.62, 3.26]	3.03 [2.56, 3.32]	3.18 [2.93, 3.45]	0.102	0.794	0.348	0.183	0.998	0.521
**NGAL**	1.78 [1.38, 2.22]	1.66 [1.36, 2.08]	2.03 [1.80, 2.56]	0.053	0.933	0.028	0.122	0.998	0.119
**Osteopontin**	4.83 [4.43, 5.32]	4.66 [4.41, 5.48]	5.54 [4.77, 6.53]	0.005	0.892	0.028	0.037	0.998	0.119
**TFF3**	4.36 [3.91, 4.82]	4.35 [3.93, 4.62]	4.61 [4.37, 4.97]	0.054	0.970	0.040	0.122	0.998	0.119

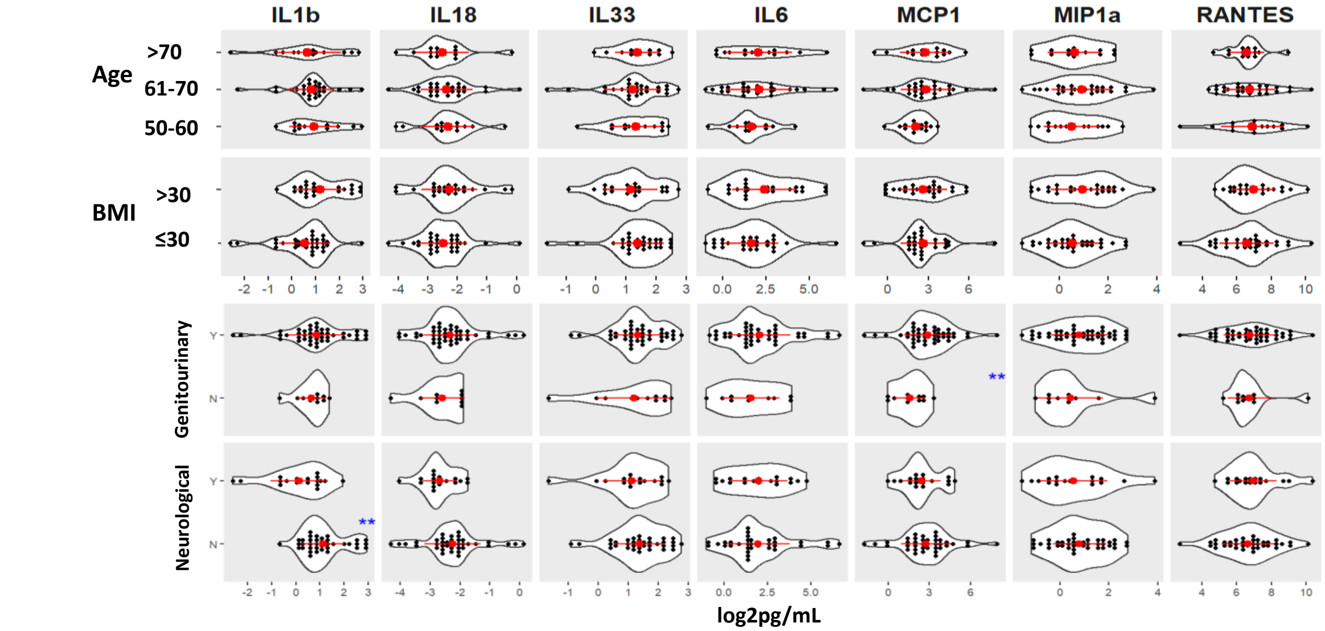

Figure S1. Cytokine levels by COVID-19 patient characteristics at baseline. *: adjusted *p*-value (FDR) < = 0.1; **: adjusted p-value (FDR) <= 0.05. Only significant variables were shown for medical history.

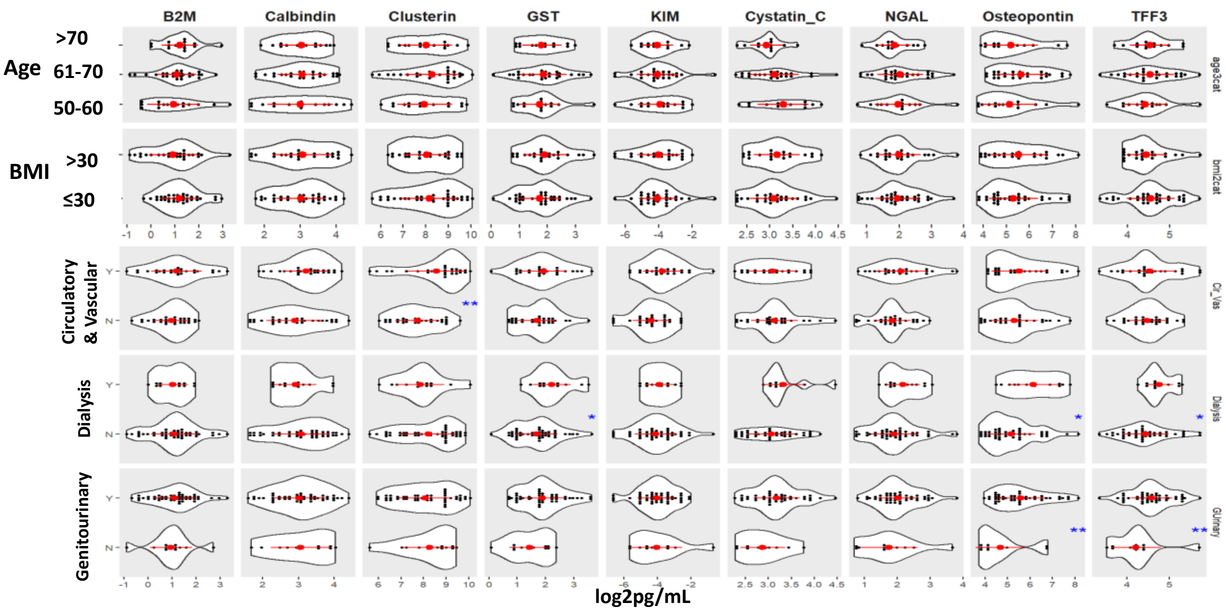

Figure S2. Renal toxicity marker levels by COVID-19 patient characteristics at baseline. *: adjusted p-value (FDR) <=0.1; **: adjusted p-value (FDR) <=0.05. Only significant variables were shown for medical history.
